# Identification of *Guiboutia* species by NIR-HSI spectroscopy

**DOI:** 10.1038/s41598-022-15719-0

**Published:** 2022-07-07

**Authors:** Xiaoming Xue, Zhenan Chen, Haoqi Wu, Handong Gao

**Affiliations:** 1 Nanjing Forest Police College, Nanjing, Jiangsu China; 2 Nanjing Forestry University, Nanjing, Jiangsu China

**Keywords:** Optics and photonics, Optical spectroscopy

## Abstract

Near infrared hyperspectral imaging (NIR-HSI) spectroscopy can be a rapid, precise, low-cost and non-destructive way for wood identification. In this study, samples of five *Guiboutia* species were analyzed by means of NIR-HSI. Partial least squares discriminant analysis (PLS-DA) and support vector machine (SVM) were used after different data treatment in order to improve the performance of models. Transverse, radial, and tangential section were analyzed separately to select the best sample section for wood identification. The results obtained demonstrated that NIR-HSI combined with successive projections algorithm (SPA) and SVM can achieve high prediction accuracy and low computing cost. Pre-processing methods of SNV and Normalize can increase the prediction accuracy slightly, however, high modelling accuracy can still be achieved by raw pre-processing. Both models for the classification of *G. conjugate, G. ehie* and *G. demeusei* perform nearly 100% accuracy. Prediction for *G. coleosperma* and *G. tessmannii* were more difficult when using PLS-DA model. It is evidently clear from the findings that the transverse section of wood is more suitable for wood identification. NIR-HSI spectroscopy technique has great potential for *Guiboutia* species analysis.

## Introduction

Wood identification is important in the modern wood industry and illegal logging monitoring. However, wood identification based on traditional technique has many disadvantages, such as low accuracy and high cost. Nowadays, DNA barcoding and GC–MS technique have a wide range of application, but these methods are time-consuming and need to know the special knowledge about wood classification. Automatic identification systems would have great advantages in fields of wood recycling and monitoring illegal logging trade in protected tree species^1^.

There are many studies on species classification based on near-infrared (NIR) spectroscopy, but few combined with hyperspectral imaging. Near infrared hyperspectral imaging (NIR-HSI) spectroscopy as a non-destructive, efficient, and high-speed modern analysis technique, can obtain both spectral and image information of the tested sample. NIR-HSI spectroscopy provides simultaneous determination of physical and chemical properties of the sample, as well as their spatial distribution, which overcoming some limitations of NIR spectroscopy. Therefore, this technique is more suitable for the analysis of heterogeneous samples and allows more reliable qualitative identification using both spectral and spatial information. Although this technique requires strong professional judgment, it has greater potential when combined with machine learning algorithms.

NIR-HSI has proved to be a reliable method to analyze soils, water, food, seeds, and other samples^2–8^. NIR-HSI has the advantages of non-invasive, timesaving, toxic-free, and suitable for unprocessed samples^9^. Very few studies have been reported about wood identification, but Te Ma et al. used near infrared spatially resolved spectroscopy (NIR-SRS) based on HSI to discriminate 15 wood samples (Softwoods: *Agathis alba, Araucaria heterophylla, Thuja plicata, Chamaecyparis obtuse, Cryptomeria japonica*, Hardwoods: *Triplochiton scleroxylon, Ochroma pyramidale, Hevea brasiliensis, Liriodendron tulipifera, Cercidiphyllum japonicum, Paulownia tomentosa, Kalopanax pictus, Fraxinus mandshurica Eusideroxylon zwageri, Fagus sylvatica*), proved that light scattering characteristics of wood can identify between 5 softwoods and 10 hardwoods^1^. Although lots of works provided encouraging results on the application of spectroscopy technique for wood identification, NIR-HSI still has great potential.

*Guiboutia* are indigenous to the Southern African region and are found in Zambia, Zimbabwe, Zaire, Namibia, and Angola^10^. Leaf and bark have been used by local indigenous for medicinal and nutritional purposes^11^. The wood is of high quality and resistant to termite and marine borer attack. Despite its hardness and heaviness, it is easy to work with and used for high class furniture, flooring, cabinet work, inlays, and railway sleepers etc. *G. demeusei* and *G. tessmannii* are known as Bubinga. Bubinga is well known for its use as a Rosewood substitute in the timber trade but has nothing in common with rosewood family. Although Bubinga is not evaluated on the IUCN (International Union for Conservation of Nature) Red List of Threatened Species, *G. demeusei* and *G. tessmannii* are listed on CITES (the Convention on International Trade in Endangered Species of Wild Fauna and Flora) appendix II., During recent years China's General Administration of Customs has reported a lot of cases related to timber illegal smuggling, and the officers are faced with the problem of difficulty in determining the level of species protection.

Bubinga and other species of the *Guibourtia* genus are highly similar in macrostructure and microstructure, no laboratory in China can provide reliable identification methods for the moment. Therefore, it is necessary to establish a non-destructive and low-cost method for the identification of *Guiboutia* species. Wood as a complex material is a combination of micro tissue and chemical substances, which are both influence the identification accuracy. Therefore, spectroscopic techniques, model type, data processing and sample handling methods are key of wood identification.

In this study, cube wood samples of five *Guiboutia* species were analyzed by NIR-HSI spectroscopy. Species were identified by PLS-DA and SVM with different data treatment methods, aims to establish a non-destructive approach for *Guiboutia* species identification, in particular two CITES-listed species. This study set out to determine whether the data pretreatment method will affect the accuracy of modeling and to assess which section of cube wood samples are suitable for species identification.

## Materials and methods

### Samples

The wood samples were taken from China National Forestry and Grassland Administration Wildlife Criminal Evidence Identification Center (Nanjing forest police college) including five *Guiboutia* species: *G. conjugate, G. ehie, G. demeusei, G. coleosperma* and *G. tessmannii.* All samples belong to criminal evidence in several illegal timber cases.

We declare that this study has the official permission to collect the plant sample and complies with the Chinese legislation. Samples were stored in China National Forestry and Grassland Administration Wildlife Criminal Evidence Identification Center; wood species have been identified by Xiaoming Xue (based on macroscopic characteristics).

Spectral data were collected from 12 trees for each species, totaling 60 trees. In this study, 5–10 air-dried samples were prepared for each tree with the dimensions of 100 mm × 100 mm × 100 mm. A total number of 318 samples were used; 212 samples were included in the training set, and 106 samples were included in the testing set. Before NIR-HSI analysis, the samples were air-dried for 45 days, and the moisture content of samples were between 9.7 and 12.4%.

### NIR-HSI spectra collecting

NIR-HSI spectra were collected using NIR-HSI spectrophotometer (ImSpectorV 10E), camera (R aptor EM285CL, UK) and 350W halogen light source (Illumination Technologies, USA). The system was operated by IR CP0076 Software (Isuzn, Taiwan). The analyses were performed within a spectral range of 982–2562 nm at 10 nm resolution, 6.2 nm wavelength intervals; the distance between the camera lens and the light source were 30 cm and 20.5 cm respectively. To reduce the generation of light shadows and obtain higher quality NIR hyperspectral images, the light source was aimed at the sample at the angle of 45°; the exposure time was set to 2.5 ms, and the delivery speed was set to 17.38 mm s^−1^. Each sample was scanned separately in transverse, radial and tangential section. After obtaining the NIR-HSI images of the samples, ROI was selected and calculated by ENVI 5.3 software (ENVI Inc., USA; URL: https://www.envi.com) as the average reflection spectrum to build models.

Image correction was performed every 45 min to minimize the interference signals, which needs scanned black and white image. Under the same conditions of sample image acquisition, the Teflon white plate (99.9% reflectance) was scanned to obtain a white image, and the camera lens was covered to obtain a black image. All the collected wood spectral images were then converted to relative reflectance values according to the following equation:

1 $$R=\frac{{R}_{0}-B}{W-B}$$

where R is the standardized light reflectance value, R_0_ donate sample reference images, W donate white reference images and B donate dark image.

### Data analysis

The datasets were processed by MATLAB R2018b (MathWorks Inc., Natick, MA, USA; URL: https://www.mathworks.com), with PLS_toolbox 802 (Eigenvector Research, Inc., Manson, WA, USA; URL: https://eigenvector.com).

NIR-HSI provides 256 spectral images at wavelengths from 982 to 2562 nm. In this study, wavelengths over 2005 nm were found to be noisy, so wavelengths from 982 to 2005 nm were put in the data set.

Spectral pretreatments may be employed to correct for the effects of instrument noise, light scattering, sample surface unevenness, and other factors on spectra and improve the performance of classification models^12^. In this study, analysis of NIR-HSI spectra data using SG (Savitzky-Golay) smoothing, SNV (Standard Normal Variate), Normalize, and MSC (Multiple scattering correction) pre-processing methods. Since the original spectrum was containing all the spectral variables and the information was redundant, SPA was selected to eliminate irrelevant or nonlinear variables. SPA replaced the original spectrum with a few key variables to reduce the amount and complexity of model operations, which can improve model stability and prediction accuracy.

Partial least squares (PLS) is a well-known statistical technique that can find the best functional match for a set of data by minimizing the sum of squares of the errors^13^. PLS is the most used regression method to identify plant species using spectroscopy data. PLS-DA is an adaptation of PLS regression methods to the problem of supervised clustering. It has seen extensive use in the analysis of multivariate datasets. PLS-DA is a versatile algorithm that can be used for predictive and descriptive modelling. This method can use for spectral analysis, which extracts latent variables and uses them to predict responses. In this study, PLS-DA models were cross-validated using Venetian blind-cross validation (5 segments) to validate the identification models.

SVM is a powerful and flexible popular machine learning tool that provides solutions for regression as well as classification problems. This technique presents a model that performs a minimization of the errors caused by outliers. SVM is effective in high dimensional spaces and still effective in cases where number of dimensions is greater than the number of samples. SVM is memory efficient, it uses support vectors in the decision function. By using this technique different Kernel functions can be specified for the decision function. In this study, SVM models were cross-validated using Venetian blind-cross validation (5 segments) to validate the classification models.

The accuracy of the model can be determined by the sensitivity, specificity, and misclassification rate. Sensitivity allows assessing how well the model can identify samples that belong to a particular class, and specificity measures the capacity of the model to reject nonbelonging samples. The misclassification rate is the ratio of false positives to the total number of samples. In this study, sensitivity, specificity, and misclassification rate were considered for evaluating the model performance.

## Results

### Spectroscopic characterization

**Figure 1:**
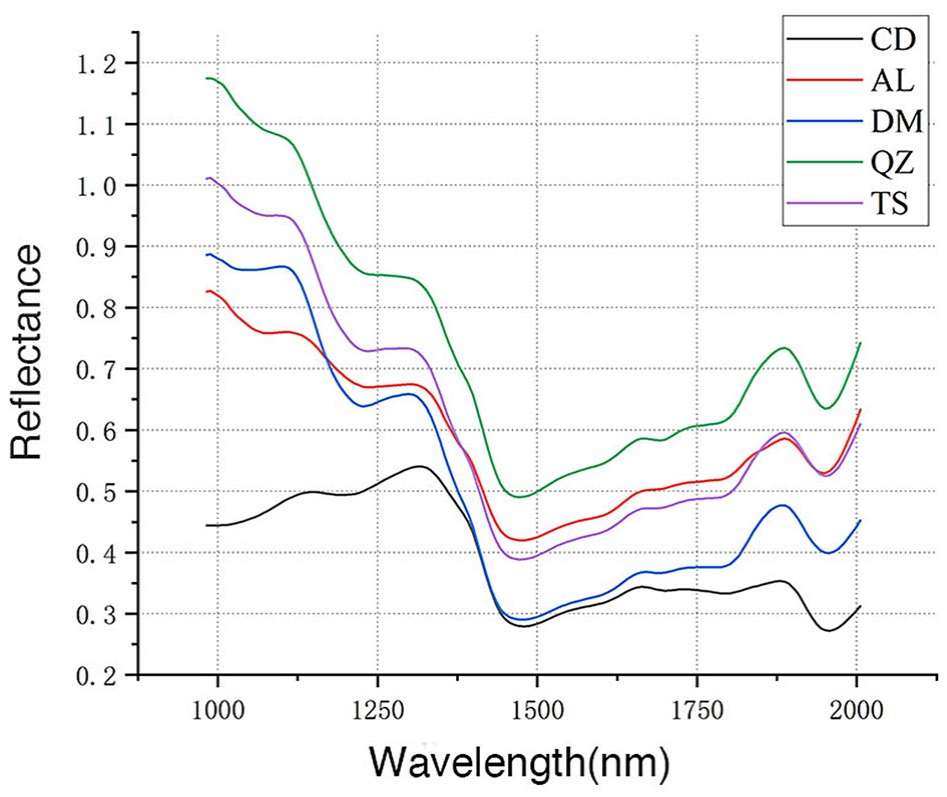
Mean spectra for the transverse section of samples. CD = *G. conjugate*; AL = *G. ehie*; DM = *G. demeusei*; QZ = *G. coleosperma*; TS = *G. tessmannii*.

**Figure 2:**
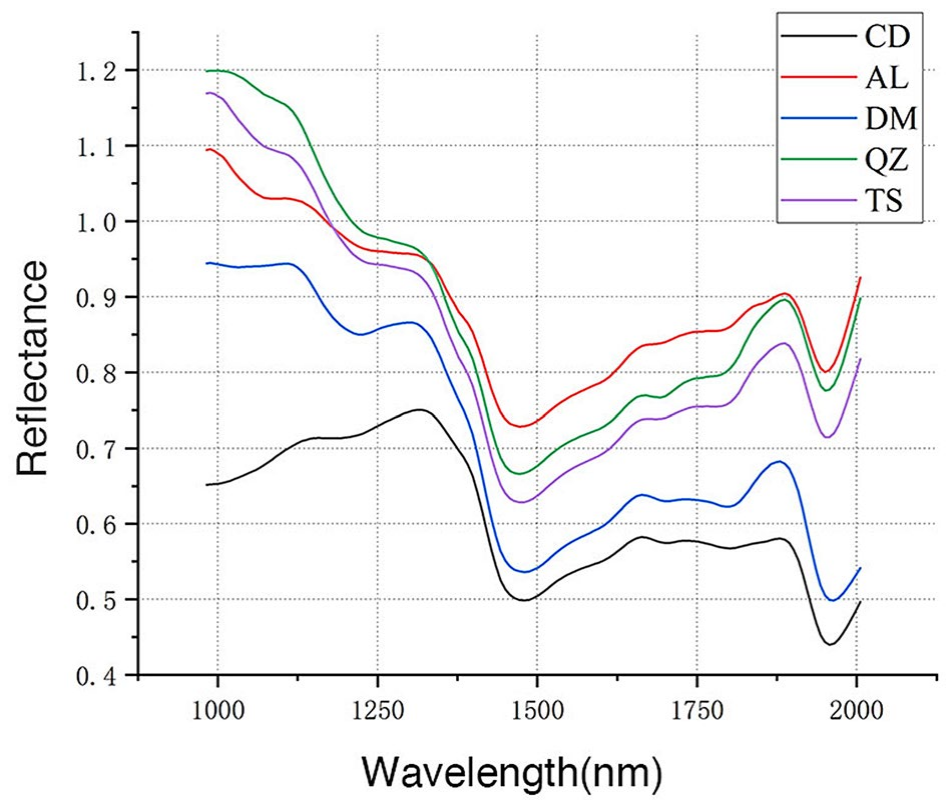
Mean spectra for the radial section of samples. CD = *G. conjugate*; AL = *G. ehie*; DM = *G. demeusei*; QZ = *G. coleosperma*; TS = *G. tessmannii*.

**Figure 3:**
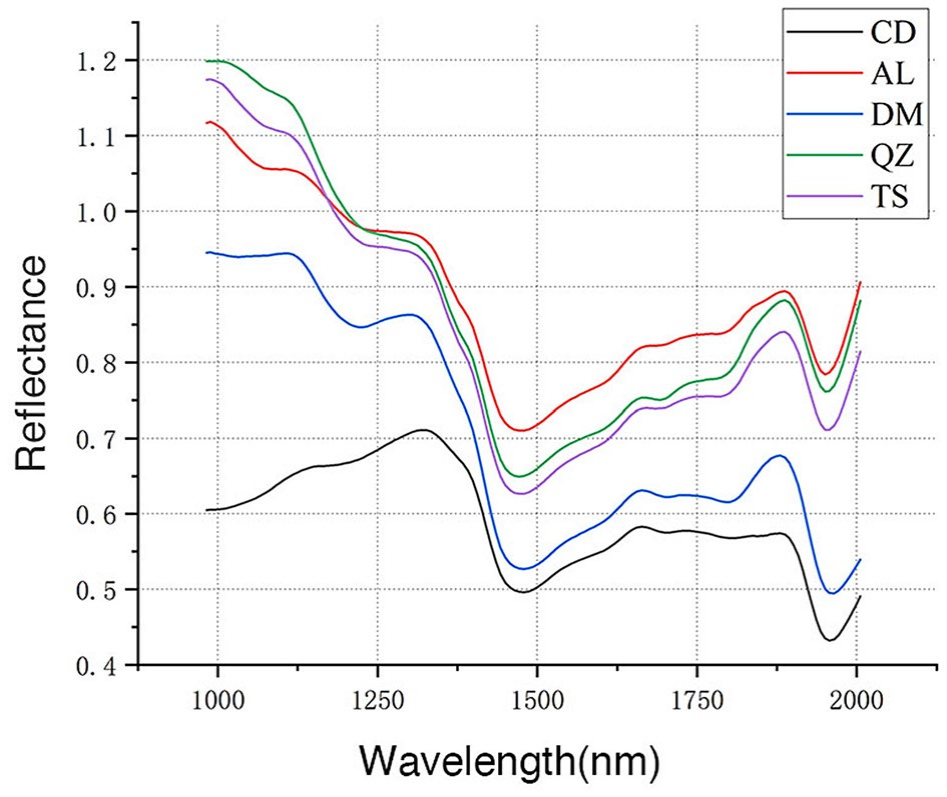
Mean spectra for the tangential section of samples. CD = *G. conjugate*; AL = *G. ehie*; DM = *G. demeusei*; QZ = *G. coleosperma*; TS = *G. tessmannii*.

Several differences between NIR-HSI spectra of five *Guiboutia* species can be observed (Figs. 1, 2, 3). It can be seen that the trends of mean spectral of different species were generally similar, except for G. *conjugate* and G. *ehie*. However, the reflectance values of each band shown significant differences because of many factors, such as geographical location, climatic factors, precipitation rate, soil fertility, etc.

It can be observed that the reflectance of G. *conjugate* was lower compared to the remaining samples, and the values shown an increasing trend within the range of 982–1312 nm. In contrast, the reflectance of G. *ehie*, G. *demeusei*, G. *coleosperma* and G. *tessmannii* shown a decreasing trend from 982 to 1471 nm and have visible absorption peaks at 1297 nm and 1887 nm.

For five species, the spectra curve of transverse section was different from radial and tangential section, while the radial and tangential section were basically the same. The mean spectral of G. *ehie* shown different trends between three wood section, it may affect the modeling results.

Due to overlapping and combination bands, only raw spectra information about spectral alterations can be provided, but a high accuracy in the wood species identification would be possible.

### PLS-DA results

The prediction results of PLS-DA are presented in Tables 1, 2, 3 and shown a high degree of accuracy. When using data obtained from transverse section, normalize provides the highest accuracy among all algorithms, while SNV and MSC pre-processing are relatively low. Different from transverse section, SNV pre-processing provides the best modelling results when processing data from tangential section. On the other hand, radial section does not need data pre-processing because the results are relatively poor.

**Table 1:** Summary of the prediction results of PLS-DA model.

	Pre-processing	Prediction accuracy		
		Transverse section (%)	Radial section (%)	Tangential section (%)
*G. conjugate*	Raw	100	100	100
	SNV	100	100	100
	SG smoothing	100	100	100
	Normalize	99.06	100	100
	MSC	100	100	100
*G. ehie*	Raw	100	100	94.34
	SNV	100	100	83.96
	SG smoothing	100	100	93.40
	Normalize	100	100	89.62
	MSC	100	100	76.42
*G. demeusei*	Raw	97.17	100	100
	SNV	84.91	100	100
	SG smoothing	100	100	100
	Normalize	98.11	100	100
	MSC	82.08	99.06	100
*G. coleosperma*	Raw	100	84.91	86.79
	SNV	100	81.13	90.57
	SG smoothing	100	82.08	87.74
	Normalize	100	79.25	87.74
	MSC	100	79.25	87.74
*G. tessmannii*	Raw	90.57	90.57	71.70
	SNV	78.30	75.47	85.85
	SG smoothing	90.57	90.57	69.81
	Normalize	93.40	92.45	72.64
	MSC	82.08	75.47	88.68

**Table 2:** Optimal wavelengths of SPA treatment.

	Optimal wavelengths (nm)	RMSE
Transverse section	1145, 1227, 1341, 1428, 1479, 1589, 1685, 1758, 1866, 1901, 1957	0.39
Radial section	1085, 1145, 1206, 1327, 1384, 1435, 1479, 1589, 1692, 1758, 1873, 1957	0.36
Tangential section	1020, 1078, 1145, 1213, 1443, 1479, 1589, 1692, 1751, 1866, 1894, 1957	0.41

**Table 3:** Summary of the prediction results of PLS-DA model (based on SPA treatment).

	Pre-processing	Prediction accuracy		
		Transverse section (%)	Radial section (%)	Tangential section (%)
*G. conjugate*	Raw	99.06	100	100
	SNV	100	100	100
	SG smoothing	99.06	100	100
	Normalize	99.06	100	100
	MSC	100	100	100
*G. ehie*	Raw	100	100	95.28
	SNV	99.06	98.11	67.92
	SG smoothing	99.06	99.06	93.40
	Normalize	100	100	88.68
	MSC	99.06	99.06	63.21
*G. demeusei*	Raw	96.23	100	100
	SNV	91.51	100	100
	SG smoothing	94.34	99.06	100
	Normalize	98.11	100	100
	MSC	89.62	100	100
*G. coleosperma*	Raw	100	86.79	87.74
	SNV	99.06	89.62	89.62
	SG smoothing	100	96.23	87.74
	Normalize	100	87.74	87.74
	MSC	99.06	88.68	88.68
*G. tessmannii*	Raw	89.62	78.30	74.53
	SNV	76.42	85.85	84.91
	SG smoothing	88.68	86.79	75.47
	Normalize	88.68	73.58	74.53
	MSC	77.36	85.85	88.68

SPA is an effective method to reduce modeling calculation. In this study, SPA algorithm has been used to reduce the number of bands. As shown in Table 5, different optimal wavelengths were selected for three sections, and radial section has the minimum RMSE value.

After SPA treatment, the prediction accuracy of PLS-DA was slightly lower than raw spectral treatment (Table 3). When processing the data obtained from transverse section, normalize pre-processing works best, but SNV and MSC were not suitable for modelling. When it comes to the data from radial section, SG smoothing pre-processing have the best modelling performance. Modelling with tangential section data do not require pre-processing, raw data will yield the best results.

The sensitivity, specificity and misclassification rate given in Table 6 were used as a measure of the classification performance of the PLS-DA model. The calculation results show that model by transverse section data has good predictive ability. In particular, the sensitivity values demonstrate that PLS-DA model after SPA pre-processing is able to correctly identify the samples (99.34% for the training set, 98.12% for the testing set), and the specificity shows that the model does not misclassify multiple times (96.12% for the training set, 96.32% for the testing set).

### SVM results

Identification of five *Guiboutia* species with SVM model was significantly better than PLS-DA model. As shown in Tables 4, 5, SVN pre-processing can achieve 100% accuracy in three sections. Different from transverse and tangential section, raw and SG smoothing pre-processing can achieve the highest accuracy when using data obtained from radial section.

**Table 4:** Summary of the prediction results of SVM model.

	Pre-processing	Prediction accuracy		
		Transverse section (%)	Radial section (%)	Tangential section (%)
*G. conjugate*	Raw	100	100	100
	SNV	100	100	100
	SG smoothing	100	100	100
	Normalize	100	100	100
	MSC	100	100	100
*G. ehie*	Raw	100	100	100
	SNV	100	100	100
	SG smoothing	100	100	100
	Normalize	100	100	97.17
	MSC	100	100	100
*G. demeusei*	Raw	100	100	100
	SNV	100	100	100
	SG smoothing	100	100	100
	Normalize	98.11	100	100
	MSC	100	100	100
*G. coleosperma*	Raw	100	100	100
	SNV	100	100	100
	SG smoothing	100	100	100
	Normalize	96.23	76.42	96.23
	MSC	100	100	100
*G. tessmannii*	Raw	99.06	100	100
	SNV	100	100	100
	SG smoothing	99.06	100	98.11
	Normalize	89.62	94.34	81.13
	MSC	100	100	100

**Table 5:** Summary of the prediction results of SVM model (based on SPA treatment).

	Pre-processing	Prediction accuracy		
		Transverse section (%)	Radial section (%)	Tangential section (%)
*G. conjugate*	Raw	100	100	100
	SNV	100	100	100
	SG smoothing	100	100	100
	Normalize	100	100	100
	MSC	100	100	100
*G. ehie*	Raw	100	100	100
	SNV	100	100	100
	SG smoothing	100	100	100
	Normalize	100	100	97.17
	MSC	100	100	98.11
*G. demeusei*	Raw	100	100	100
	SNV	100	100	100
	SG smoothing	99.06	100	100
	Normalize	97.17	100	100
	MSC	100	100	100
*G. coleosperma*	Raw	100	100	100
	SNV	100	100	99.06
	SG smoothing	100	100	100
	Normalize	99.06	100	95.28
	MSC	100	100	99.06
*G. tessmannii*	Raw	100	100	100
	SNV	99.06	100	100
	SG smoothing	97.17	100	97.17
	Normalize	91.51	96.23	94.34
	MSC	97.17	100	100

In this study, SPA algorithm was applied when SVM model constructing. SVM has a huge calculating cost, but SPA can reduce it significantly. As shown in Table 6, modelling after SPA does not need data pre-processing, the best performance was obtained using raw data. The misclassification rate was low when using transverse section data, indicating that the developed model can be used for classification purposes (0.09% for training set and 0.00% for testing set, Table 6).

**Table 6:** Summary of the quality statistical parameters of PLS-DA and SVM model (transverse section).

Model	Pre-processing	Tests	Sensitivity (%)	Specificity (%)	Misclassification rate (%)
PLS-DA	–	Training	99.34	95.80	2.17
		Testing	99.44	95.98	2.45
	SPA	Training	99.34	96.12	2.74
		Testing	98.12	96.32	3.02
SVM	–	Training	99.82	99.96	0.19
		Testing	99.82	99.96	0.19
	SPA	Training	99.90	99.98	0.09
		Testing	100.00	100.00	0.00

### Comparison of modelling performance between three sections

**Figure 4:**
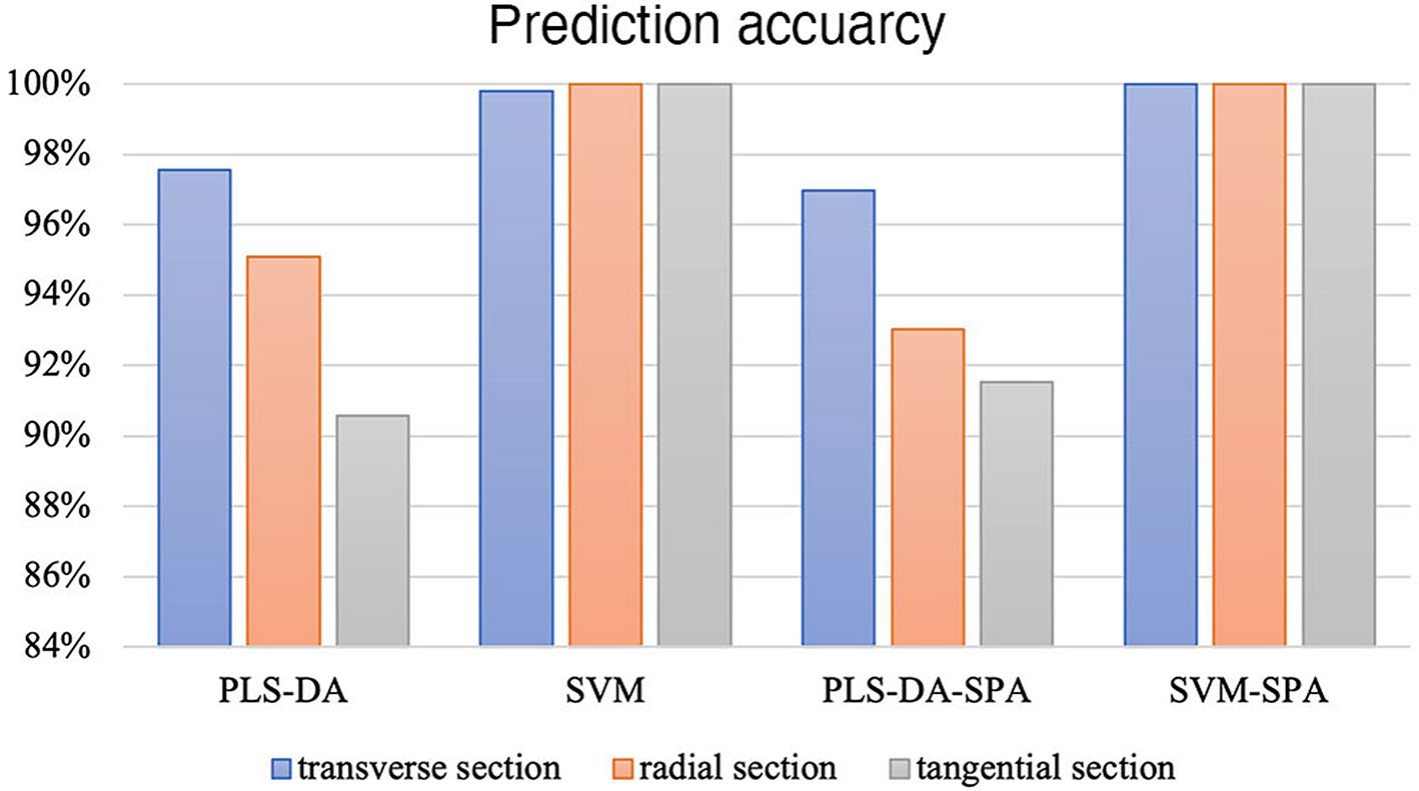
Model prediction accuracy of three sections.

In this study, transverse, radial and tangential section spectral were used for model constructing. As shown in Fig. 4, PLS-DA modelling of data from transverse section shown the highest accuracy (97.55%), while prediction accuracy was relatively low with tangential section (90.57%). But a significant improvement in identification accuracy was reached by tangential section after combined with SPA treatment (96.98%).

When it comes to SVM, the prediction accuracy of three sections were basically the same. Raw spectral treatment modelling results can achieve 100% except for transverse section (99.81%), and after SPA treatment the accuracy can all reaching 100%.

In general, when modelling with PLS-DA, using transverse section could achieve satisfactory performance, while transverse section was undesirable. On the other hand, data from all section are suitable for modelling with SVM method.

### Mixing matrix

**Figure 5:**
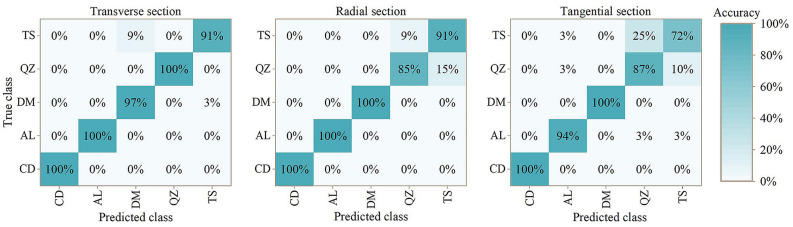
Mixing matrix for results of five *Guiboutia* species with PLS-DA model. CD = *G. conjugate*; AL = *G. ehie*; DM = *G. demeusei*; QZ = *G. coleosperma*; TS = *G. tessmannii*.

**Figure 6:**
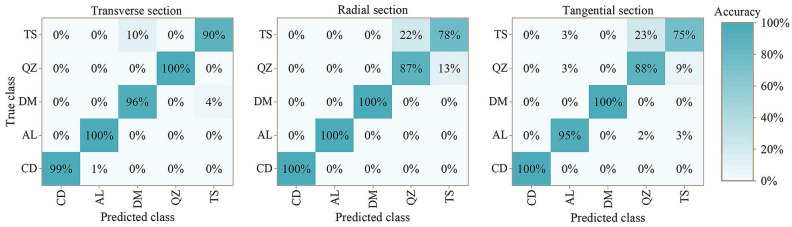
Mixing matrix for results of five *Guiboutia* species with PLS-DA model (based on SPA spectral treatment). CD = *G. conjugate*; AL = *G. ehie*; DM = *G. demeusei*; QZ = *G. coleosperma*; TS = *G. tessmannii*.

Mixing matrix can reflect the classification accuracy of models. Figure 5 shows the classification results of PLS-DA model. Figure 6 shows the classification results of PLS-DA model based on SPA treatment. G. *conjugate* can be classified with 100% accuracy except for transverse section based on SPA treatment (99%). In addition, G. *ehie* and G. *demeusei* shown results with high accuracy. When it comes to G. *coleosperma* and G. *tessmannii*, the classification results were comparatively low, while the best results were obtained in transverse section.

**Figure 7:**
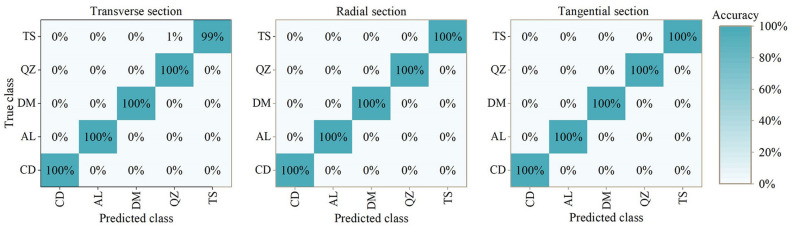
Mixing matrix for results of five *Guiboutia* species with SVM model. CD = *G. conjugate*; AL = *G. ehie*; DM = *G. demeusei*; QZ = *G. coleosperma*; TS = *G. tessmannii*.

**Figure 8:**
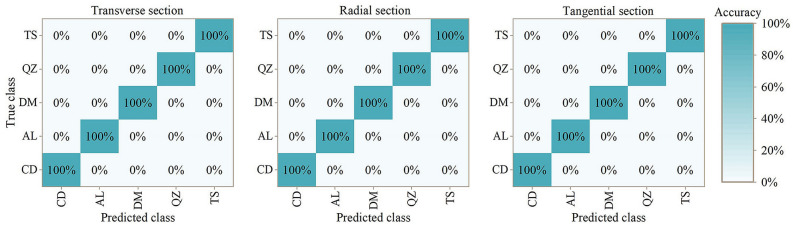
Mixing matrix for results of five *Guiboutia* species with SVM model (based on SPA spectral treatment). CD = *G. conjugate*; AL = *G. ehie*; DM = *G. demeusei*; QZ = *G. coleosperma*; TS = *G. tessmannii*.

Figure 7 shows the classification results of SVM model, and Fig. 8 shows the results based on SPA treatment. It can be concluded that SVM model have reliable classification performance. Modelling with data from three sections based on SPA treatment have 100% accuracy.

## Discussion

### Spectroscopic characterization

In general, the NIR-HSI spectra of five *Guiboutia* species have a slight difference, presenting systematic variations of the baseline. The wavelength outside 982 to 2005 nm did not provided any important information, because there are so many noises which affect the performance of models. Reducing the wave number range has a significant effect on improving the classification results of the wood samples.

It is obviously that there is a substantial difference between the spectra of three sections (Figs. 1, 2, 3), it may relate to the higher accuracy of modelling with data obtained from transverse section. As shown in Figs. 1, 2, 3, the spectra of *G. coleosperma* and *G. tessmannii* were similar, so it is difficult to distinguish these two species using NIR-HSI.

Spectroscopic characterization can reflect both the physical and chemical properties of wood^14^ and sensitive to moisture content of samples^15^. Further studies can be combined with chemometrics to achieve a more accurate identification of five *Guiboutia* species by chemical contents.

### NIR-HSI spectra data pre-processing

In this study, for the sake of suppressing the unfavorable influence brought by noise, SG smoothing, SNV, Normalize, and MSC pre-processing were employed to analyze the NIR-HSI spectra data.

SG smoothing is an effective spectral pre-processing method with a wide range of application and a variety of different modes^16^. The number of smoothing points is an important parameter. If the smoothing points set was too little, it would cause errors. If the points set too much, the spectra information would be lost. So unsuitable smoothing points number would decrease the accuracy of model, select reasonable number of points is necessary^17^. In this study, 15 smoothing points were been selected for spectra data pre-processing, which get high model accuracy. In this study, models using SVN show low reliability, only perform well when constructing PLS model based on SPA with radial section data.

SNV and Normalize are classic pre-processing method for scatter correction of NIR data, both methods do not involve a least-square fitting in their parameter estimation, they can be sensitive to noisy entries in the spectrum^18^. In this study, models using SNV and Normalize provide better accuracy than other methods. As shown in Tables 1, 2, 4, 5, SNV exhibited high accuracy when construct SVM models.

MSC is a widely used spectral pretreatment method. MSC can remove imperfect data from the matrix before modelling. MSC have two steps, including correction coefficients estimation and recorded spectrum correcting. In this study, MSC did not improved the accuracy of models and even reduced it.

Overall, when identifying five *Guiboutia* species, spectra data pre-processing did not improve the accuracies of models significantly. As shown in Tables 1, 2, 4, 5, the SVM model using raw pre-processing can achieve 100% accuracy after SPA treatment, this method can be considered as a robust way to modelling.

### SPA treatment

NIR-HSI can provide spectral information over a large number of wavelengths for each sample. But in many cases, NIR-HSI instrument have information redundancy when getting data, which lead the increased workload and even models unreliable. Variable selection techniques can used to improve the prediction and parsimony ability of multivariate calibration models. SPA is a variable-selection technique that has attracted increasing interest in the analytical-chemistry community within the past 10 years, this method was originally be used in Multiple Linear Regression (MLR) models^19^.

In this study, several optimal wavelengths were selected for model constructing and shown credible performance with RMSE of 0.39, 0.36 and 0.41 for transverse section, radial section, and tangential section respectively. Transverse section spectral were selected 11 optimal wavelengths which related to the fastest modelling speed and reliable prediction accuracy. For SVM model, a slight improvement of the identification ability of the model can be observed after SPA pre-processing. The sensitivity value increased from 99.82 to 99.90%, the specificity value increased from 99.96 to 99.98%, and the misclassification rate decreased from 0.19 to 0.09%. As compared to the transverse section, radial and tangential section need more optimal wavelength numbers but did not reduce the error, so modelling is not recommended. SPA model for *Guiboutia* species, developed with a small number of wavelengths, showed that a simpler model is able to predict the types of wood.

### Models results

In this study, the results suggested a high degree of accuracy of two different classification model for spectral data, but modelling speed are significantly difference. Overall, SVM have higher accuracy, but greater computational cost. SPA based on SVM can considerably increase the speed of modelling and not reduce the reliability of models, so this method is suitable for five *Guiboutia* species identification.

Spectral technology can use branch, leaf, bark, and trunk to identify the species of trees^1,20–22^. The use of trunk samples for analysis in NIR-HSI may provide some advantages compared to other part of trees, because these parts are generally more variable than trunk. Trees at different development stages may differ in chemical composition, and cell construction, so the spectral data of samples could change^20,23^. On the other hand, trunk is not susceptible to be contaminated by bacteria and fungi compared other parts, so the spectral properties of samples are not easy be to modify^24^. This study used transverse, radial, and tangential section of trunk to construct model and shown high identification accuracy. Relatively speaking, transverse section performed better, and tangential section are not suitable for identification.

Among the five tree species, *G. conjugate, G. ehie and G. demeusei* are easy to be classified, but *G. coleosperma* and *G. tessmannii.* are more likely to be confused due to similar physical properties of wood and place of origin. As shown in Figs. 3, 4, 5, 6, SVM have better performance to classify five *Guiboutia* species, and PLS-DA performed relatively worse.

## Conclusions

In general, NIR-HSI spectroscopy combined with SPA treatment and SVM models was confirmed to be an alternative for non-destructive identification method for five *Guiboutia* species, and transverse section is the most suitable surface for model construction.

The SPA treatment has proved to be a reliable method to improve model performance. The real advantage of this technology is the possible to develop a dedicated spectrophotometer device or a faster portable device for wood identification, due to the low number of optimal wavelengths. These devices can improve the accuracy of *Guiboutia* species identification and reduce the detection cost significantly.

Transfer from laboratory to production implementation and illegal logging monitoring needs further investigation into the influence of sample surface variations and wood moisture content to the identification results.

Summarizing, we demonstrated that it is possible to identify *Guiboutia* species using NIR-HSI spectroscopy with high accuracy, the main advantage of this technique is fast, precise, cheap, non-destructive, and own broad application prospects in forestry related areas.

## Data Availability

The datasets generated and analyzed during the current study are available in the Figshare repository, [10.6084/m9.figshare.19673547.v1].
